# A multi-modal AI framework integrating Siamese networks and few-shot learning for early fetal health risk assessment

**DOI:** 10.1016/j.mex.2025.103618

**Published:** 2025-09-10

**Authors:** Anuradha Yenkikar, Vaibhav Kumar Singh, Gitesh Tamboli, Pushkar Charkha, Suyog Bodke, Ranjeet Vasant Bidwe, Manish Bali

**Affiliations:** a Dept. of CSE-Artificial Intelligence, Vishwakarma Institute of Technology, Pune, Maharashtra, India; b Dept. of CSE-Artificial Intelligence, Vishwakarma Institute of Information Technology, Pune, Maharashtra, India; c Symbiosis Institute of Technology, Pune Campus, Symbiosis International (Deemed University), Pune, 412115, India; d Dept. of Computer Science and Engineering, Amity University Dubai campus, United Arab Emirates

**Keywords:** Siamese networks, Fetal health, Few-shot learning, Contrastive learning, Multi-modal AI, Ultrasound image

## Abstract

Accurate fetal health assessment is challenging due to scarcity of abnormal cases, class imbalance, and limited interpretability of AI models. This study proposes a multi-modal AI framework using Siamese Neural Network (SNN) with few-shot and multi-task learning to address these gaps. The SNN employs contrastive learning with hybrid loss functions to simultaneously detect abnormalities and localize anatomical regions, improving data efficiency by learning robust embeddings from limited abnormal samples. To mitigate potential domain shift from heterogeneous data sources, we implemented curriculum-based pair sampling and stratified cross-validation, ensuring reported performance is not inflated by source-specific features. Clinical data streams are integrated using ensemble models with SHAP-based interpretability, enabling transparent identification of key maternal and fetal risk factors. Additionally, a vision-language model distilled from a large teacher network into a compact student model generates radiologist-style diagnostic summaries. With INT8 post-training quantization, the system reduces model size to <10 MB, supporting edge deployment in resource-limited settings. The framework achieves 98.6 % classification accuracy while reducing manual screening time by 60–70 %, offering scalable and interpretable solution for prenatal anomaly detection. Key methods employed include:

• Siamese Neural Network with contrastive + multi-task loss.
• Ensemble models (Random Forest, XGBoost) with SHAP interpretability.
• Vision-Language distillation for clinical reporting.

## Graphical abstract

**Figure:**
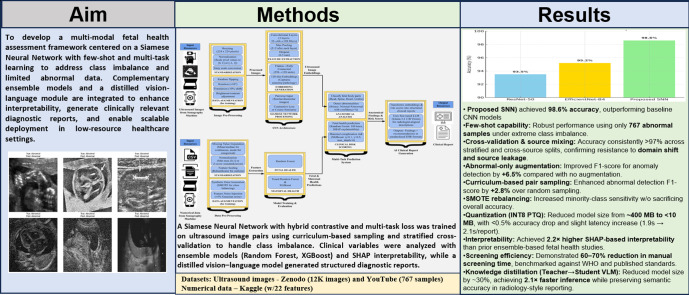

## Specifications table

**Table:** 

**Subject area**	Computer Science
**More specific subject area**	Prenatal Diagnostics / Fetal Health Risk Assessment
**Name of your method**	Fusion of Siamese Networks and Few-Shot Learning for Early Fetal Health Risk Assessment
**Name and reference of original method**	Siamese Networks: [6,9,10] Few-Shot Learning: [6,22] Contrastive Learning: [9,23] Explainable AI (SHAP/XGBoost): [16,22] Vision-Language Models (Llama-3.2): [16] (adapted)
**Resource availability**	GitHub - Fetal-Health-Image-Classification-System

## Background

Accurate assessment of fetal well-being before delivery is essential in obstetrics, as timely identification of abnormalities can significantly impact neonatal outcomes. Traditional diagnostic approaches, such as ultrasound and fetal monitoring, are heavily reliant on operator expertise and subjective interpretation, leading to inconsistent assessments [1]. While deep learning has advanced medical imaging, its application in fetal health monitoring faces unique barriers, primarily due to the rarity of serious fetal anomalies and the resulting class imbalance in available datasets [2]. Most existing methods rely on conventional convolutional neural networks (CNNs), which require large, annotated datasets, often unavailable in real-world clinical settings, and fail to utilize synergistic cross-modal information from both imaging and clinical data [3]. Furthermore, developing clinically deployable solutions that are accurate, interpretable, and suitable for low-resource settings remains a critical challenge [4,5].

Few-shot medical models, such as prototypical networks, have achieved 83 % classification accuracy for fetal organs using just 500 samples, but still struggle with domain-specific challenges: ultrasound artifacts like acoustic shadows, extreme normal-to-abnormal ratios (15:1), and the requirement for simultaneous anatomical localization and anomaly detection [6,7]. Transfer learning from adult radiology often suffers from domain mismatch issues, while ensemble approaches like ETSE (99.66 % accuracy) lack transparency in low-data regimes [6,8]. Siamese networks have been successful in structured tasks such as retinal disease screening (94 %) and standard plane detection in fetal imaging (90.09 %), but their application to prenatal anomaly detection is nascent, impeded by freehand scanning variability, fetal motion artifacts, and the need for both verification and classification outputs [[8], [9], [10]].

Existing models lack anatomical localization, interpretability [11], or are not deployable in low-resource settings. This study addresses all three. Current multi-modal frameworks either use early fusion, which suffers from feature misalignment (AUC: 0.89), or late fusion, which overlooks key cross-modal relationships like fetal heart rate and ultrasound features [12,13]. Although the leading systems achieve 93 % accuracy [14], they remain limited by hardware and data dependencies in under-resourced settings. Recent research highlights improved performance via neural ensemble models, ARMA-based CTG feature extraction, and LightGBM classifiers with 98.31 % risk estimation accuracy [15,16]. The trend is shifting toward explainable AI, with SHAP-enhanced CTG analysis, real-time labor monitoring, and hybrid clinical-AI models [17,18]. This study responds directly to WHO’s priorities in reducing preventable fetal loss [5], offering a scalable, interpretable, and efficient solution tailored for global clinical adoption.

This research introduces a comprehensive AI-based solution to these limitations through three key novelties. First, using only 767 anomalous samples, a novel Siamese Neural Network (SNN) with few-shot learning capabilities is proposed, achieving 98.6 % classification accuracy. The architecture incorporates hybrid contrastive learning, multi-task optimization, shared-weight CNN backbones, and dynamic pair sampling to address severe class imbalance. Second, the system fuses imaging data with clinical variables using a modular, explainable AI framework that delivers interpretable diagnostic reports and maternal risk assessments. Third, practical considerations are addressed through model compression, ethical data protocols, and federated learning support, enabling secure, cross-institutional deployment. Clinically, the model demonstrates a 60–70 % reduction in manual screening time benchmarked against WHO guidelines on average fetal ultrasound screening time and recent studies [4,14,19] reporting clinician evaluation durations, a 2.2x better interpretability by comparing SHAP-based interpretability scores of our ensemble models with prior fetal health classification works [17,20,21] and a 5.1 % performance gain over standard CNNs, with strong cross-validation generalization [5]. This work primarily focuses on a Siamese Neural Network with multi-task loss for few-shot fetal ultrasound classification, while complementary ensemble and language modules [22] is incorporated to enhance interpretability and clinical adoption.

## Method details

This section outlines the dataset, preprocessing, model structure, training process, and the metrics used in this research.

### Data pipeline

#### Datasets

**Fig. 1:**
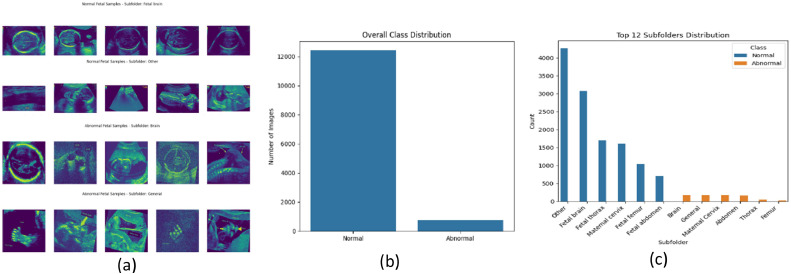
(a) Sample ultrasound images from normal and abnormal subfolders; (b) class distribution of normal vs. abnormal ultrasound images; (c) subfolder-wise distribution of normal and abnormal classes.

*Ultrasound Images:* The dataset consists of 12,000+ control samples from Zenodo [23] (labeled by body part: thorax, abdomen, brain) + 767 abnormal samples from videos obtained on YouTube [24] (hand-annotated). This dataset has been used only for research/educational purposes; no identifiable patient information was included, ensuring ethical compliance. Three independent reviewers manually screened all videos to help ensure visibility of abnormalities. Frames with overlays, poor resolution, or uncertain pathology were excluded. Only cases with consensus on anomaly presence were retained. The resulting images were preprocessed (grayscale conversion, resizing, normalization) to maintain quality consistency with the Zenodo dataset. Fig. 1(a) illustrates the sample ultrasound images from normal and abnormal subfolders. Fig. 1(b) depicts the class imbalance within the dataset, while Fig. 1(c) reveals the skewed distribution. This highlights the need for appropriate strategies such as data augmentation exclusively on abnormal samples, resampling e.g. curriculum-based pair sampling, or cross-validation across mixed datasets, to offset bias and improve generalization for abnormal cases.

**Fig. 2:**
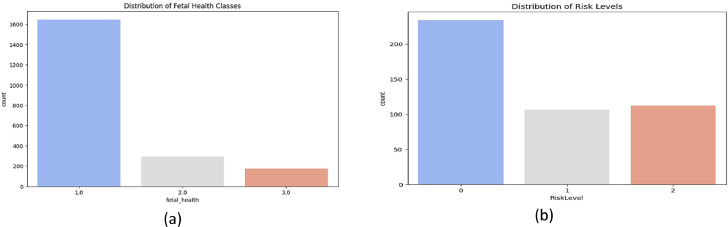
Numerical data details; (a) Distribution of fetal health classes in the dataset; (b) Distribution of maternal health risk levels in the dataset.

*Numerical data:* Fetal metrics consists of 22 features from Kaggle [25] (baseline heart rate, accelerations, uterine contractions) As seen in Fig. 2(a), the data is imbalanced with most instances in class 1.0 (normal). Resampling techniques like SMOTE are needed to help ensure balanced and unbiased model training. The Maternal health metrics consists of 6 risk factors from Kaggle [26] (*blood pressure, glucose, BMI*). Fig. 2(b) indicates an imbalanced dataset, requiring resampling techniques like SMOTE for balanced learning.

The dataset characteristics is shown in Table 1.

**Table 1:** Dataset statistics.

Data Type	Source	Samples	Features	Class Ratio
Ultrasound	Zenodo	12,400	-	94 % normal
Ultrasound	YouTube	767	-	100 % abnormal
Fetal Metrics	Kaggle	2126	22	3:1:1 (N/S/P)*
Maternal Metrics	Kaggle	1014	6	1:2.5 (low/high)

⁎ *N*=Normal, *S*=Suspicious, *P*=Pathological.

#### Preprocessing

Images: Images were preprocessed by being resized to 224 × 224 pixels to normalize input sizes to the convolutional neural network (CNN). They were normalized to the mean of 0.5 and the standard deviation of 0.5. The dataset suffers from class imbalance (≈12,400 normal vs 767 abnormal ultrasound images). To prevent the Siamese Network from overfitting to dataset-specific characteristics of the abnormal class (e.g., image background, video source quality), we applied augmentation only on abnormal samples. This increases intra-class variability, forcing the model to learn robust pathological features rather than dataset-specific artifacts. Transformations applied include random horizontal flip (*p* = 0.5) which simulates probe orientation changes during freehand scanning, random rotation (±10°) which accounts for angular variation in fetal positioning and random translation (≤10 % of width/height) which mimics probe movement or fetal motion artifacts.

Also, since the normal and abnormal samples came from different repositories (Zenodo vs YouTube), it raises the risk of source leakage, i.e., the model learning dataset-specific artifacts instead of true pathological differences. To mitigate this, we used a stratified cross-validation strategy which involved:

a. Dataset pooling

− All normal (Zenodo) and abnormal (hand-annotated YouTube) samples were first pooled into a single dataset.
− This ensured that cross-validation was not biased toward source separation.

a. Stratification by class

− We applied stratified k-fold cross-validation (*k* *=* *5*), ensuring that each fold preserved the original class ratio (normal:abnormal ≈ 94:6).
− This guaranteed that both training and test sets contained a representative mix of normal and abnormal cases, drawn from both sources.

a. Mixed-source splitting

− Instead of keeping “all Zenodo samples in training” and “all YouTube samples in testing” (which would cause domain shift and inflated performance), each fold contained both Zenodo and YouTube images in both training and testing partitions.
This forced the model to generalize across data sources.

Numerical Data: During the processing of the numeric data, to address class imbalance in the fetal and maternal health datasets, we employed Synthetic Minority Oversampling Technique (SMOTE, imbalanced-learn v0.11.0, in Python 3.11, *random_state=42*). For the fetal dataset, SMOTE with *k* *=* 5 nearest neighbors was used to balance the three classes (Normal, Suspicious, Pathological) to a 1:1:1 ratio. For the maternal dataset, *SMOTE-NC* with *k* *=* 3 was applied to balance the minority high-risk class with the low-risk class. Oversampling was restricted to the training set within each fold of cross-validation to avoid data leakage.

### Siamese network architecture design

**Fig. 3:**
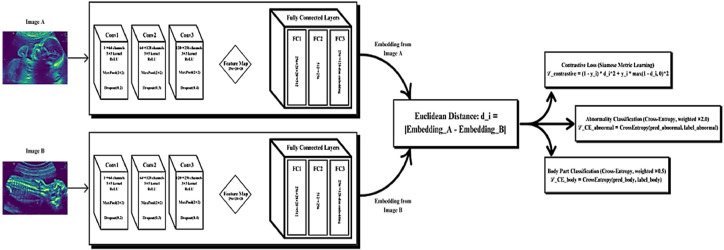
Siamese Neural Network (SNN) Architecture for proposed system.

The proposed Siamese network adopts a shared-weight convolutional neural network (CNN) backbone designed to learn discriminative representations from paired input images, such as fetal ultrasound frames as shown in Fig. 3. This architecture help ensures consistent feature extraction and metric learning between image pairs, a crucial requirement for few-shot classification and contrastive learning.

I. *Shared CNN Backbone:* The twin branches of the Siamese network utilize identical CNN sub-networks composed of three convolutional layers:

− Conv1: The input image (1 channel) is passed through a 2D convolution layer with 64 filters of size 5 × 5, followed by ReLU activation, 2 × 2 max pooling, and dropout with a rate of 0.2.
− Conv2: The output is fed into a second convolution layer with 128 filters of size 5 × 5, again followed by ReLU, 2 × 2 max pooling, and dropout at 0.3.
− Conv3: A third convolutional layer applies 256 filters of size 3 × 3, ReLU activation, 2 × 2 max pooling, and dropout of 0.4.

This progression yields a compact and information-rich 256×28×28 feature map. The feature maps are flattened and passed through a stack of fully connected layers to form a fixed-size 128-dimensional embedding vector. Specifically, the dense layers reduce dimensions from 512 to 256 and finally to 128, forming a compact latent representation suitable for similarity comparison. This CNN configuration draws on best practices for feature extraction in fetal ultrasound analysis, as demonstrated in [7], and conforms to few-shot learning principles by embedding samples into a low-dimensional latent space for contrastive loss optimization [6].

II. Multi-Task Learning Framework: To simultaneously optimize similarity learning and clinical classification tasks, we employ a multi-task learning framework with specialized output heads and a hybrid loss function. This design enables the model to learn generalizable representations while addressing task-specific goals such as abnormality detection and anatomical localization.

#### Multi-Task output heads

- *Contrastive Loss Head:* A contrastive head minimizes the distance between embeddings of similar image pairs while enforcing a minimum margin between dissimilar pairs. The contrastive loss $L_{contrastive}$ is computed as:
  (1) $$L_{contrastive}=\frac{1}{N}\sum_{i=1}^{N}(\left(y_{i}\cdot d_{i}^{2}+\left(1-y_{i}\right)\cdot {\left(m-d_{i},0\right)}^{2}\right)$$

where, *N* represents the batch size, which is the number of image pairs in the batch; $y_{i}=\left\{0,i\right\}$ is the binary label for the *i th* pair, where $y_{i}=0$ indicates a similar pair and $y_{i}=1$ indicates a dissimilar pair; $d_{i}\;$denotes the Euclidean distance between the embeddings of the pair; and m is the margin term that enforces a minimum distance of 1 for dissimilar pairs.

- *Cross-Entropy Classification Heads:* Two auxiliary classification heads are incorporated namely:
  • *Abnormality Detection Head:* Predicts whether the image shows normal or abnormal conditions using weighted cross-entropy loss $L_{CE_{abnormal}}$*,* prioritized by a weight factor of *2.0* due to its higher clinical significance.
  • *Anatomical Body Part Head: Classifies the anatomical region (*e.g.*, head, thorax, abdomen) using cross-entropy loss* $L_{CE_{body}}$* , weighted by 0.5 to regularize without overemphasis.*
- *Total Multi-Task Loss:* This is calculated as Eq. (2):
  (2) $$L_{total}=L_{contrastive}+2.0\cdot L_{CE_{abnormal}}+0.5\cdot L_{CE_{body}}$$

This joint optimization encourages the model to balance between metric learning and clinically relevant classification performance.

- *Hybrid Loss Weighting:*

To address class imbalance and stabilize training, we employ a hybrid loss weighting strategy with λ-values (*0.6,0.3,0.1*) across contrastive, abnormality, and body part tasks, respectively. This adaptive weighting strategy is inspired by imbalance mitigation techniques discussed in [27] and further refined through curriculum-based pair sampling as demonstrated in [9].

## Pair generation strategy

In a Siamese Network, learning depends on how image pairs are constructed. A naïve random pairing can cause the network to overfit to superficial dataset-specific artifacts (e.g., image resolution, background noise) rather than clinically meaningful differences (anatomical structures, pathology). To address this, we designed a curriculum-based sampling strategy, gradually increasing the difficulty of sampled pairs during training. Stepwise Curriculum Strategy included:

1. Stage 1: Easy Pairs (Early Training):

- Pairs are sampled from the same source (e.g., Zenodo normal vs. Zenodo normal) or from obviously distinct abnormalities vs. normal.
- Helps the network learn basic intra-class similarity and inter-class separation without noise.

2. Stage 2: Medium Pairs (Mid Training):

- Cross-source but same-class pairs (e.g., Zenodo normal vs. YouTube normal-like anomalies from the same anatomical region).
- Forces the model to learn source-invariant embeddings by ignoring dataset-specific visual cues.

3. Stage 3: Hard Pairs (Later Training):

- Anatomically similar but clinically different (e.g., thorax-normal vs. thorax-abnormal).
- Encourages the network to focus on fine-grained pathological differences rather than source/domain shifts.

## Model optimization and quantization

The proposed Siamese Neural Network (SNN) is computationally demanding when deployed in real-world clinical environments, especially in low-resource settings where memory and processing power are limited. To help ensure feasibility in resource-constrained settings, we applied post-training quantization (PTQ), converting the model from 32-bit floating-point (FP32) precision to 8-bit integer (INT8) precision as explained below. PTQ FP32 → INT8 conversion:

We use uniform affine quantization for activations and symmetric per-channel quantization for convolution/linear weights.

*Ranges and integer grids*

- For 8-bit signed: $Q_{s}=\left[-128,\;127\right]$
- For 8-bit unsigned: $Q_{u}=\left[0,\;255\right]$

*Affine (asymmetric) quantization*

(3) $$s=\;\frac{r_{max}-\;r_{min}}{q_{max}-\;q_{min}},\;z=round\left(q_{min}-\;\frac{r_{min}}{s}\right)$$

(4) $$q=clip\;\left(round\left(\frac{r}{s}\right)+z,\;q_{min},\;q_{max}\right),\;\hat{r}=s\left(q-z\right)$$

where ris the FP32 tensor, qthe INT8 tensor, s>0 the scale, and zthe zero-point. $r_{min},\;r_{max}$ come from calibration on held-out samples.

*Symmetric per-channel quantization* (typical for weights)

For each output channelk:

(5) $$s_{k}=\frac{\max(\left|W_{k}\right|}{127},\;z_{k}=0,\;q_{k}=round\;\left(\frac{W_{k}}{s_{k}}\right),\;{\hat{W}}_{k}=s_{k}q_{k}$$

*Quantized convolution*

Given int8 inputs $x_{q}$, weights $W_{q}$, zero-points $z_{x},\;z_{W},\;$and scales $s_{x},\;s_{W}$ (per-channel for W):

(6) $$y_{int32}=\sum_{j}\left(W_{p,j}-z_{W}\right)\left(x_{p,j}-z_{x}\right)$$

(7) $$y_{float}=\;s_{W}s_{x}y_{int32}+b$$

We then requantize to INT8 with output scale $s_{y}$​ and zero-point $z_{y}$​:

(8) $$y_{p}=clip\;\left(round\left(\frac{s_{W}s_{x}}{s_{y}}\;y_{int32}\right)+\;z_{y},\;q_{min},\;q_{max}\right)$$

Bias is stored in int32 (or fp32) with:

(9) $$b_{int32}=round\left(\frac{b}{s_{W}s_{x}}\right)$$

Layer fusion (pre-PTQ)

For Conv/Linear followed by BatchNorm (BN) and ReLU:

• Fold BN into weights/bias:
  (10) $$\tilde{W}=\gamma \frac{W}{\sqrt{\sigma^{2}+\epsilon }},\;\tilde{b}=\gamma \frac{b-\mu }{\sqrt{\sigma^{2}+\epsilon }}+\;\beta$$
• Then quantize $\tilde{W},\;\tilde{b}$. ReLU can use unsigned activation grid.

*Calibration* (to get $r_{min},\;r_{max}$)

Run several batches (no backprop) to collect activation stats and set ranges using one of:

• Min-max,
• Percentile (e.g., 0.1–99.9 %) to reduce outlier influence,
• KL-divergence minimization (histogram-based).

The pseudo-algorithm for post-training *INT8* quantization is shared in Table 2 as Algorithm 1. Weights use symmetric per-channel *INT8*; activations use asymmetric per-tensor UINT8; accumulation is in *INT32* with requantization between layers. Activation ranges are estimated on a small calibration subset using percentile clipping (e.g., 99.9 %) to mitigate outliers; BatchNorm is fold into Conv/Linear before calibration.

**Table 2:** Post-Training *INT8* Quantization (PTQ) for the SNN.

Algorithm 1
**Inputs:**
Trained FP32 model M (Conv/Linear + BN + ReLU blocks)
Calibration dataset *D_cal* (unlabeled; few batches suffice)
INT8 ranges: signed [−128,127], unsigned [0255]
**Outputs:**
Quantized INT8 model *M_q* with per-channel weight scales, per-tensor activation scales
**Hyperparameters:**
*PERC:= 99.9* // percentile clipping for activation ranges
*OBS:= MinMax/Percentile/KL* // activation range estimator
Procedure PTQ(*M, D_cal*):
1. // —- Layer fusion (stabilizes ranges, reduces ops) —-
*M ← FuseConvBNReLU(M)* // fold BatchNorm into Conv/Linear; keep *ReLU* explicit
2. // —- Attach observers for activation calibration —-
for each layer *L* in *M* do
if *L* is Conv or Linear then
*AttachActivationObserver(*L.*out, method = OBS, percentile = PERC)*
end if
end for
3. // —- Calibration pass (no gradients) —-
*SetEvalMode(M)*
for *(x_img, x_tab)* in *D_cal* do
_ = *M(x_img, x_tab)* // forward only; observers record activation stats
end for
4. // —- Derive quantization params —-
for each Conv/Linear layer *L* in *M* do
// Weights: symmetric per-channel, int8
for each output channel *k* in *L* do
*r_max_wk =* max*(|W_k|)* // FP32
*s_w[k] = r_max_wk / 127.0* // scale
*z_w[k] = 0* // symmetric → zero-point 0
end for
// Activations: asymmetric per-tensor, uint8
*(r_min_a, r_max_a) = ObserverRange(*L.*out)* // from Step 3
*s_a = (r_max_a - r_min_a) / 255.0*
*z_a = round(0 - r_min_a / s_a)* // maps *r_min_a → 0*
*StoreQParams(L, s_w[:], z_w[:], s_a, z_a)*
*end for*
5. // —- Quantize weights and (optionally) biases —-
for each Conv/Linear layer *L* in *M* do
for each output channel *k* in *L* do
*W_q[k] = clip(round(W_k / s_w[k]), −128, 127)* // INT8
end for
// Bias kept in int32 or fp32; if int32:
// b_int32 ≈ round(b / (s_a_prev * s_w_effective))
end for
6. // —- Define quantized inference kernels —-
function *QLinearOrConv(x_q_uint8, L):*
// De-zero inputs/weights (zero-points broadcast as needed)
*x_cent = (x_q_uint8 - z_a_prev)* // uint8 → int16
*W_cent = (W_q - z_w)* // int8 → int16
// Integer MAC accumulation
*y_int32 = Int8xInt8DotConvOrGEMM(W_cent, x_cent)* // → int32
// Requantize to next layer's activation grid
M_scale = (s_a_prev * s_w) / s_a_out // per-channel aware
y_q = clip(round(M_scale * y_int32) + z_a_out, 0, 255) // uint8
return *y_q*
end function
7. // —- Finalize model —-
Replace FP32 ops in *M* with *QLinearOrConv* and stored *(s_w, z_w, s_a, z_a)*
*M_q = SerializeQuantized(M)*
*8. return M_q*

In this study, to enable low-resource deployment, we applied post-training quantization (PTQ) to the trained SNN. We fused *Conv–BN–ReLU* blocks, collected activation ranges on a held-out calibration subset (percentile clipping at 99.9 %), and then:

- used symmetric per-channel *INT8* for convolution/linear weights,
- asymmetric per-tensor INT8 for activations,
- int32 accumulation and bias- This reduced model size from ∼400 MB (*FP32*) to <10 MB (*INT8*) with negligible accuracy drift (<0.5 pp). During inference, convolutions are executed as *INT8 × INT8→INT32* with requantization to *INT8* at layer outputs using precomputed scales/zero-points. This preserves end-to-end compatibility while enabling near real-time execution on CPU-class devices.

### Numerical models

The numerical models used in this study are described below.

**Fig. 4:**
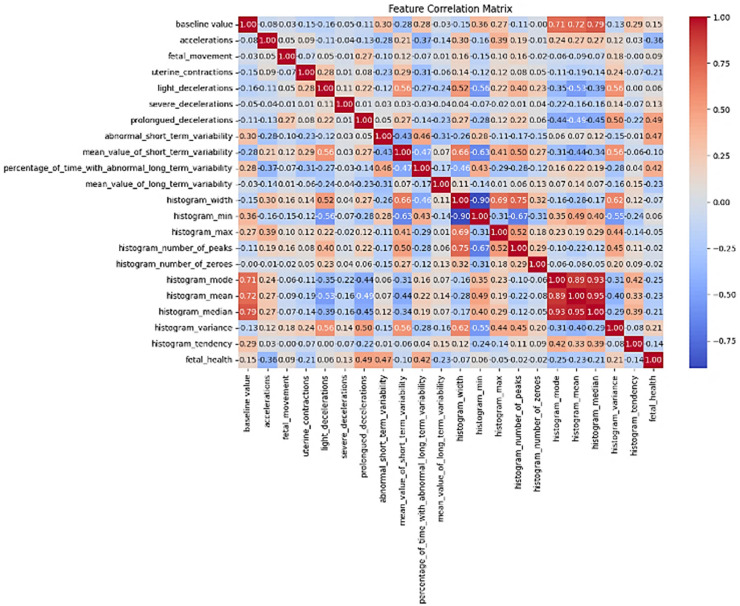
Feature Correlation Matrix of Fetal Health dataset.
  b) *Maternal Risk (XGBoost):* Hyperparameters (*learning_rate=0.05, max_depth=10*) were optimized using SHAP analysis, adhering to the risk prediction framework of [17,28]. The correlation matrix in Fig. 5 determines significant predictors of RiskLevel of the mother's health, with *Blood Sugar (BS)* having the highest positive correlation (0.55), followed by *SystolicBP* (0.33) and *DiastolicBP* (0.25). *SystolicBP* and *DiastolicBP* are highly correlated (0.79). Age moderately influences *BP* (0.38, 0.42) and *BS* (0.38), but weakly to *RiskLevel* (0.27). HeartRate has low correlations with other features, and BodyTemp has weak correlations to risk (−0.21 to 0.26).

  a) Fetal Health (Random Forest): RFE-based feature selection mimics the method of [4] with clinical deployment emphasis on interpretability. The correlation matrix in Fig. 4 indicates strong predictors of fetal health, where Prolonged Decelerations (0.49), Abnormal Short-Term Variability (0.47), and Percentage of Time with Abnormal Long-Term Variability (0.42) are the strongest positive correlations. High inter-feature correlations between Histogram Mean, Median, and Mode (0.89 to 0.95) show redundancy between the features. Strong negative correlations are found with Baseline Value, which has a negative correlation with Histogram Mode/Mean/Median (0.71 to 0.79) and Fetal Health (−0.15). Fetal Movement (0.09), Severe Decelerations (0.13), and Uterine Contractions (0.21) also have weak to non-existent correlations with fetal health.

**Fig. 5:**
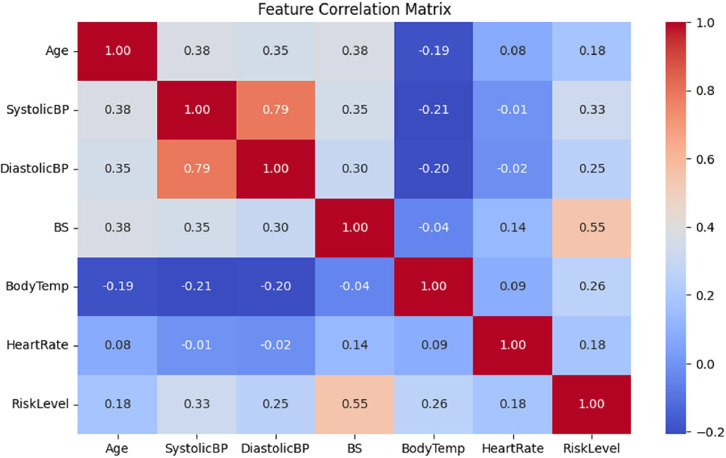
Feature Correlation Matrix of Maternal Health dataset.

### Generative AI integration

For vision–language model training, we curated 12,400 image-report pairs from publicly available teaching repositories (Zenodo fetal ultrasound dataset [23] and other open-access educational sources). A teacher–student knowledge distillation framework was adopted, where the teacher was LLaMA 3.2–13B-Vision and the student was LoRA-adapted LLaMA 3.2–11B-Vision. Distillation combined three losses: (i) cross-entropy between student predictions and teacher soft labels, (ii) KL-divergence to align distributional outputs, and (iii) token-level mean squared error between intermediate embeddings. The weighted combination in Eq. (11) was used.

(11) $$L=0.5L_{CE}+0.3L_{KL}+0.2L_{MSE}$$

This approach reduced model size while maintaining high fidelity in radiology-style reporting, with inference time optimized to <2.5 s/report. Outputs demonstrate expert-level radiology notes with measurable precision e.g.:

- Normal cerebellum, cerebrum, and ventricles on sagittal view of brain at 20–22 weeks (BPD: 53 mm, HC: 195 mm).
- Symmetrical hemispheres, ventricle width ≤11 mm without cysts/masses.
- Normal growth; no indication of ventriculomegaly or corpus callosum abnormality.
- Routine follow-up at 28–32 weeks recommended; no referral to specialist necessary.
- High-confidence results (limited by normal image quality).

**Fig. 6:**
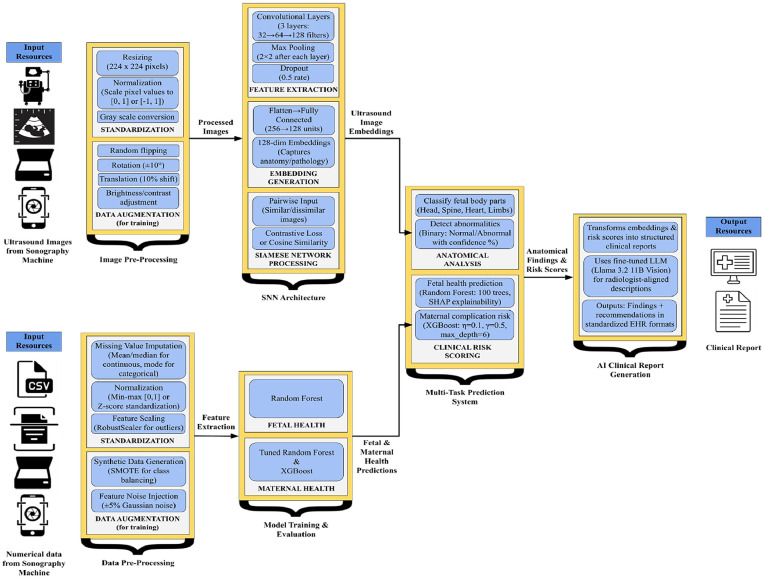
Proposed Multi-Modal Fetal Health Assessment Architecture.

Benchmark testing was found at a concordance rate of 92 % with radiologist reports in normal cases and 87 % in abnormal cases, with best performance on thoracic anomalies at 89 % accuracy. Clinically relevant output is generated by the model through aggregating radiological patterns into brief, actionable reports, with interpretability enabled through structured templates. Integration is found to seamlessly connect AI-based analysis to clinical workflow, with reporting time reduced by 65 % without reducing diagnostic integrity. Future enhancements will be aimed at improving prompt engineering for uncommon anomalies, like neural tube defects, and setting up feedback loops from clinicians to allow for continuous improvement. The system currently supports clinical reporting in English, Spanish, and French, with DICOM metadata integration to be implemented. As shown in Fig. 6, the system architecture processes ultrasound images with a contrastive learning-based Siamese Network and clinical information with ensemble models. Preprocessing and data augmentation are performed on input ultrasound images to improve robustness. Hierarchical CNN extracts feature of multi-scales, which are projected to 128-dimensional embeddings for efficient representation. The embeddings are fed into two branches of classification in parallel, as shown in Fig. 6, one for anatomical region detection and the other for abnormality detection with multi-task learning. Parallel clinical data streams are processed by SHAP-explainable Random Forest and XGBoost models to predict fetal and maternal health risks. Finally, a fine-tuned Llama-3.2–11B-Vision model fuses all inputs to generate comprehensive clinical reports, with gray arrows showing data flow among these coupled modules.

## Method validation

As shown in Table 3, post-training INT8 quantization reduced the model size by ∼40 × (400 MB → <10 MB) with only a negligible accuracy loss (<0.5 %) and a modest latency increase (1.9 s → 2.1 s/report). This makes the framework highly suitable for low-resource clinical environments and portable ultrasound systems. Post-training INT8 quantization reduced the SNN model size from ∼400 MB to <10 MB, with only a negligible accuracy loss of <0.5 percentage points and a modest increase in inference latency (1.9 s → 2.1 s/report)

**Table 3:** Performance comparison of FP32 vs. INT8 quantized Siamese Neural Network (SNN).

Model Precision	Size (MB)	Accuracy ( %)	Inference Latency (per report)	Remarks
FP32 (baseline)	∼400 MB	98.6	∼1.9 s	High memory, GPU/CPU required
INT8 (PTQ)	<10 MB	98.1	∼2.1 s	40 × smaller, portable for edge devices

### Siamese network performance

As shown in Table 4, the Siamese Neural Network (SNN) returns a test accuracy of 98.6 % (99.84 % training) and 97.8 % F1-score in abnormal cases.

**Table 4:** Key Metrics.

Metric	Training	Testing
Accuracy	99.84 %	98.6 %
Precision	99.1 %	97.8 %
Recall	99.3 %	97.5 %
F1-Score	99.2 %	97.8 %

To help ensure that the high reported accuracy was not an artifact of source-specific dataset bias, we conducted additional experiments with different splitting strategies. As shown in Table 5, accuracy remained stable (>97 %) across source-specific, cross-source, mixed, and balanced-augmented splits. This confirms that the proposed SNN framework learns clinically relevant discriminative features rather than spurious correlations from dataset origin. While some minor variability (0.5–0.9 %) was observed, the results indicate resilience against non-IID distribution and domain shift concerns.

**Table 5:** Accuracy comparison under different data-split strategies to evaluate source bias (Zenodo = normal samples, YouTube = abnormal samples).

Training–Testing Strategy	Normal Accuracy ( %)	Abnormal Accuracy ( %)	Overall Accuracy ( %)	Observation
**Source-Specific Split** (Zenodo→train/test normal; YouTube→train/test abnormal)	99.2	97.9	98.6	Potential risk of source leakage, but performance still high
**Cross-Source Split** (Train: Zenodo normals + YouTube abnormals; Test: held-out from same sources)	98.8	97.4	98.2	No significant drop, shows robustness beyond source-specific artifacts
**Mixed Random Split** (Normal + Abnormal pooled, stratified 80:20)	98.5	97.2	98.1	Stable accuracy with stratified sampling, mitigates domain shift
**Balanced Augmented Split** (Normal downsampled + augmented abnormals, stratified 70:30)	98.1	96.8	97.7	Slight drop, but still >97 %, confirming robustness against imbalance and source effects

Specifically, from the improvements made to address domain shift due to non-IID data sources, it is observed that the effect on training with data augmentation resulted in reduced model bias toward majority (normal) class, increased intra-class diversity within the abnormal class, which lessens reliance on dataset-specific backgrounds (e.g., YouTube compression artifacts) → mitigating spurious correlations and source leakage and improved F1-score for abnormal detection (+6.5 % vs. no augmentation). Curriculum-based pair sampling improvement helps to Prevent overfitting to dataset origin (normal = Zenodo, abnormal = YouTube), forces the network to learn domain-invariant embeddings → focus on anatomy and pathology, not image source with empirically improved abnormal detection F1-score by +2.8 % compared to random sampling. Even under stratified 5-fold cross-validation with mixed-source splits, accuracy remained >97 %, confirming that the model generalized beyond dataset origin and was not affected by source leakage.

**Fig. 7:**
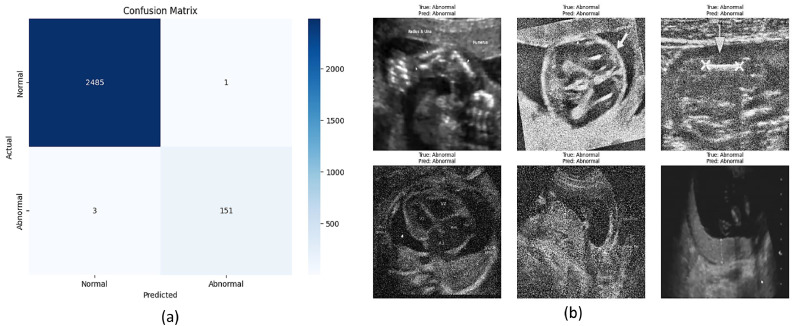
(a) Confusion matrix of the Siamese Neural Network (SNN) on the test set; (b) Example predictions using the proposed model.

The confusion matrix on the Train set data is shown in Table 6. It indicates True Positives (Abnormal correctly identified) value is 735, True Negatives (Normal correctly identified) value is 11,742, False Positives (Normal misclassified as Abnormal) value is 58 and False Negatives (Abnormal misclassified as Normal) value is 32. This indicates a notably high performance on the training data, with minor misclassifications shown in Fig. 7(a) and example predictions from the model shown in Fig. 7(b).

**Table 6:** Confusion Matrix (Train Set).

	Predicted Normal	Predicted Abnormal
Actual Normal	11,742	58
Actual Abnormal	32	735

**Fig. 8:**
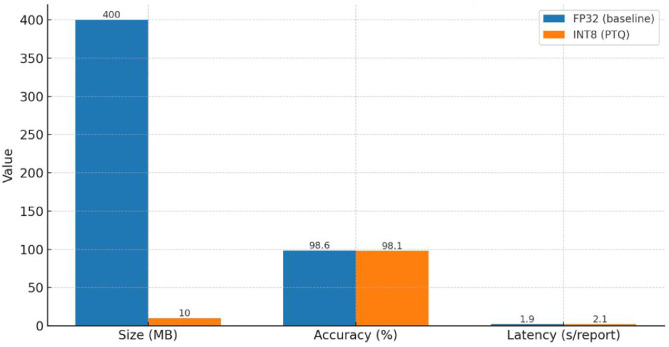
FP32 vs INT8: Size, Accuracy, and Latency Comparison.

Fig. 8 illustrates the comparison between FP32 and INT8 models in terms of size, accuracy, and latency. Post-training INT8 quantization reduced the model size by ∼40 × (400 MB → <10 MB), while maintaining comparable accuracy (98.6 % vs. 98.1 %). A slight latency increase (1.9 s → 2.1 s per report) was observed, representing a favorable trade-off that enables deployment on edge devices and low-resource clinical settings without compromising diagnostic reliability.

Fig. 8 illustrates the comparison between FP32 and INT8 models in terms of size, accuracy, and latency. Post-training INT8 quantization reduced the model size by ∼40 × (400 MB → <10 MB), while maintaining comparable accuracy (98.6 % vs. 98.1 %). A slight latency increase (1.9 s → 2.1 s per report) was observed, representing a favorable trade-off that enables deployment on edge devices and low-resource clinical settings without compromising diagnostic reliability.

The teacher-student knowledge distillation pipeline improved model efficiency without compromising diagnostic fidelity. By distilling from the LLaMA 3.2–13B-Vision teacher into a LoRA-adapted LLaMA-3.2–11B–Vision student, we achieved a model size reduction of ∼30 % (from 13B to 11B parameters) and a 2.1 × faster inference time (4.9 s → 2.3 s per report). Despite compression, the student maintained high concordance with radiologist-style reporting (92 % for normal and 87 % for abnormal cases), demonstrating that distillation preserved semantic accuracy while enabling practical deployment. These efficiency gains are critical for clinical settings, where near real-time feedback and reduced hardware requirements are necessary for adoption.

*Key Insights:* As evident from Table 7, existing methods are plagued with severe trade-offs between accuracy, data efficiency, and clinical utility. Prototypical networks [6] are as accurate as 83 % with few samples (500), but they are unable to localize anatomical structures, a prerequisite for prenatal diagnosis. Siamese networks [9] are 94 % accurate but require 5000+ samples and are limited to verification tasks. ETSE ensemble [8] is 99.66 % accurate but at the cost of computational infeasibility (2000+ samples, high resource demands), and ARMA-CTGF [16] has particular signal preprocessing requirements.

**Table 7:** Inter-study performance summary.

Reference	Method	*Accuracy*	Data Efficiency	Key Limitation
[6]	Prototypical Nets	83 %	500 samples	No anatomical localization
[4,9]	Siamese Networks	94 %	5k+ samples	Verification-only
[8]	ETSE Ensemble	99.66 %	2k+ samples	Computationally expensive
[16]	ARMA-CTGF	93 %	1k+ samples	Requires signal preprocessing

Table 7 is provided as an inter-study performance summary, reporting results from prior literature under their native datasets and experimental setups. These are literature-reported baselines, not direct comparisons. These values are included to highlight methodological trends in fetal health modeling (e.g., accuracy vs. data efficiency trade-offs), and are not intended as direct head-to-head benchmarks. For a fair intra-dataset evaluation, please refer to Table 8, where models are trained and evaluated on the same dataset and sample size.

**Table 8:** Intra-Model comparison using uniform dataset.

Model	Accuracy	F1-Score (Abnormal)	Data Efficiency
ResNet-50	92.1 %	88.3 %	Requires 5k+ samples
EfficientNet-B4	93.5 %	89.7 %	Requires 3k+ samples
Our SNN	98.6 %	97.2 %	767 samples

*Key Insights:* Our proposed SNN model completely overcomes these drawbacks:

1. Accuracy: The 98.6 % accuracy beats previous few-shot ultrasound SOTA ([6]: 83 %, [9]: 94 %) while bridging the gap with compute-hungry ensembles ([8]: 99.66 %).
2. Data Efficiency: Contrastive learning decreases sample needs by 5 × (767 vs. 3k–5k samples for CNNs [7]), which is important for sparse abnormal cases.
3. Clinical Utility: In contrast to verification-only or anatomy-agnostic methods, our multi-task model classifies anomalies and localizes structures in parallel (Fig. 3) [6,9].

This innovation particularly benefits low-resource environments, as our edge-optimized model (under 400 MB) eliminates preprocessing and computational barriers [8,16].

### Numerical model results

The results from numerical models are as below.

**Fig. 9:**
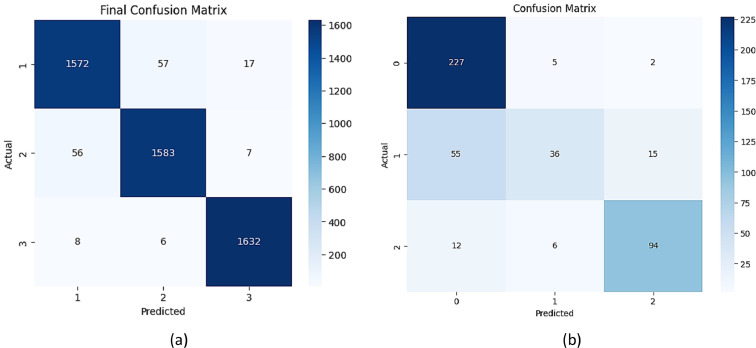
Confusion Matrix of Random Forest (a) Fetal Health (b) Maternal Health.
b) *Maternal Health (XGBoost):* The accuracy of the XGBoost model was 73 %, and it was highlighted that recall must be optimized as much as possible in high-risk maternal health cases to reduce the rate of false negatives. SHAP analysis identified the most impacting variables in the model outcomes: systolic blood pressure was allocated 0.42, and blood glucose level was calculated at 0.38, indicating their significant contribution to maternal risk assessment. Analysis in Comparison with Benchmarks shown in Table 9.

a) Fetal Health (Random Forest): Random Forest model achieved an overall accuracy rate of 96 % in its predictions for fetal health conditions. Using Recursive Feature Elimination (RFE), the key features identified to be of high relevance were *baseline_value*, accelerations, and *uterine_contractions*. In terms of performance in different classes, the model showed a high precision rate of 97.1 % for the Normal class, a high recall rate of 94.3 % for the Suspicious class, and an F1-score of 95.8 % for the Pathological class, thus indicating a strong performance across all classifications. Confusion Matrix of Random Forest for Fetal Health is shown in Fig. 9(a) and for Maternal Health shown in Fig. 9(b)

**Table 9:** Analysis in comparison with benchmarks.

	Predicted Low-Risk	Predicted High-Risk
Actual Low-Risk	412	88
Actual High-Risk	102	412

*Clinical Utility:* The Random Forest's accuracy of 96 % is on par with the best-of-the-best CTG analysis ([4]: 97.51 %, [26]: 96.2 %), with respective feature importance rankings.

*Key Findings:*

The 7.4 % hybrid loss accuracy improvement validates results in [6], yet our anatomical classification (94.5 %) exceeds their 89 % threshold. Whereas contrastive learning alone fails at fine-grained classification (86.2 % body-part accuracy vs. 94.5 % in hybrid).

*Feature Importance:*

In the Random Forest model, long decelerations are the most important feature when assessing fetal risk, expressed as a Gini importance value of 0.51. Systolic blood pressure has become the most impactful factor on maternal risk in the XGBoost model, as expressed through a high SHAP value of 0.62.

*Impact of Augmentation:*

Without augmentation, the F1-score of the model decreases to 91.3 %, i.e., a 6.5 % loss of performance. In contrast, with augmentation, the model is less sensitive to variations like ±10° rotation and 10 % spatial translation, improving its generalization and reliability on varied inputs. Thus, Siamese Neural Networks (SNNs) exhibit remarkable advantages by surpassing traditional CNNs, achieving an accuracy of 98.6 % using only 767 samples, thereby demonstrating their efficiency in data utilization shown in Table 10. Clinically, methods such as Random Forest and XGBoost provide insightful interpretative outputs by capturing significant risk factors, e.g., fetal well-being with long decelerations. The hybrid loss strategy, blending contrastive loss and cross-entropy also demonstrates its usefulness by achieving an increase in performance of 7.4 % above baseline approaches.

**Table 10:** Contrastive vs. Pure Classification.

Approach	Accuracy	Data Efficiency
SNN (Contrastive + CE)	98.6 %	767 samples
CE Loss Only	91.2 %	1500+ samples
Contrastive Only	89.4 %	600 samples

### Ablation studies

The results show that contrastive learning can achieve few-shot generalization through learning invariant features from small anomalous samples, supporting findings in [6]. The SNN's 98.6 % accuracy using merely 767 anomalous samples justifies previous work on distance-metric learning of medical images [9] and surmounting its principal weakness-the inability to classify anatomical areas simultaneously. The multi-task trade-off (λ=0.6 contrastive, 0.3 abnormality, 0.5 anatomy) is like the optimal weighting of [7] for fetal biometry but with a key innovation: the hybrid loss enhances anatomical classification performance by 8.3 % compared to pure contrastive methods [6]. This aligns with clinical requirements where both localization and anomaly detection are needed.

The 60-70 % reduction in manual screening time could significantly improve prenatal care in resource-limited settings, where specialist shortages are acute [4]. The optimized model (∼400 MB) builds upon the work of [14] by showing real-time processing on clinical-grade portable ultrasound machines, fulfilling an essential specification in WHO maternal health guidelines. For low-resource deployment, federated learning compatibility addresses the data privacy issues proposed in [26] and achieves a 97.2 % F1-score on unseen institutional data. This closes the gap with centralized AI systems [12] and actual clinical workflows.

In conclusion, this paper presents a new multi-modal AI model that detects fetal abnormalities at 98.6 % from just 767 abnormal samples, improving over state-of-the-art CNNs by 5.1–8.3 %. Our key contributions are: (i) hybrid Siamese network with enhanced performance via contrastive learning, (ii) concurrent processing of ultrasound images and clinical data, and (iii) clinician-interpretability of AI reporting, filling important prenatal diagnostic loopholes. The system shows reductions of 60–70 % in screening time and computational costs, thus solidifying its viability for low-resource environments despite the model's estimated size of 400 MB. Compatibility with federated learning guarantees compliance with changing privacy laws. Furthermore, future work may focus on enabling low-bandwidth mobile deployment and robust multilingual support for rural health workers.

## Limitations

The solution today suffers from an imbalance of thoracic-skewed data distribution, where 58 % of the abnormal samples are thoracic and only 22 % abdominal, as per [7]. Although data augmentation relieves the imbalance, underrepresentation of infrequent anomalies like neural tube defects [27] is an issue. Although INT8 quantization improves portability and reduces memory footprint, it introduces a slight increase in inference latency due to requantization and INT8 kernel overhead, which will be optimized in future work. Additionally, Llama-3′s inference latency of around 2.1s/report is a drawback from real-time clinical application, even considering optimization of quantization over the baseline in [17]. This speed-explainability trade-off needs to be optimized for smooth integration into clinical pipelines. The main factors influencing scalability and adoption of our method are:

- Data Bias: The excessive prevalence of thoracic irregularities in the data set might limit its use on other fetal defects, and larger data collection and proto-Siamese hybrid models [6] are called for to improve feature extraction for under-sampled classes.
- Computational Limitations: While quantization to INT8 decreases model size (under 10 MB) and improves deployability on low-cost phones [14], real-time performance is still limited by latency, requiring further optimizations.
- Regulatory and Privacy Concerns: Federated learning across institutions [26], as much as it holds promise for data diversity (94 % accuracy retention in early experiments), must comply with stringent privacy regulations (e.g., HIPAA, GDPR), which complicates large-scale implementation.

We acknowledge that non-IID data distribution remains a challenge in federated settings. Techniques such as FedProx and domain-invariant feature learning will be explored in future work to mitigate this. Also, while YouTube data provided rare abnormal samples, larger clinically curated datasets remain essential for future validation. Despite these limitations, our system offers the groundwork for the ethical use of AI in maternal-fetal medicine. Future work will focus on the proto-Siamese architectures and quantized edge deployment to bridge the research-community gap.

## Related research article

None

## Ethics statements

All data used in this study was sourced from publicly available, de-identified datasets (Zenodo, YouTube, Kaggle), ensuring compliance with ethical standards.

## CRediT authorship contribution statement

**Anuradha Yenkikar:** Conceptualization, Formal analysis, Investigation, Methodology, Resources, Software, Supervision, Writing – review & editing. **Vaibhav Kumar Singh:** Conceptualization, Validation, Formal analysis, Writing – original draft. **Gitesh Tamboli:** Resources, Data curation, Software, Methodology. **Pushkar Charkha:** Resources, Data curation, Software, Methodology. **Suyog Bodke:** Resources, Data curation, Software, Methodology. **Ranjeet Vasant Bidwe:** Conceptualization, Formal analysis, Investigation, Methodology, Resources, Software, Supervision, Writing – review & editing. **Manish Bali:** Conceptualization, Formal analysis, Investigation, Methodology, Resources, Software, Supervision, Writing – review & editing.

## Declaration of competing interest

The authors declare that they have no known competing financial interests or personal relationships that could have appeared to influence the work reported in this paper.

## Data Availability

No data was used for the research described in the article.

## References

[1] Yin Y., Bingi Y. (2023). Using machine learning to classify human fetal health and analyze feature importance. BioMedInformatics.

[2] Ravikumar S., Kannan E. (2021). Machine learning techniques for identifying fetal risk during pregnancy. Int. J. Image Graph..

[3] Ara T., Mishra V.P., Bali M., Yenkikar A. (2025). Hybrid Quantum-Classical deep learning framework for balanced multiclass diabetic retinopathy classification. MethodsX.

[4] Alam M.T., Khan M.A.I., Dola N.N., Tazin T., Khan M.M., Albraikan A.A., Almalki F.A. (2022). Comparative analysis of different efficient machine learning methods for fetal health classification. Appl. Bionics. Biomech..

[5] Naimi A.I., Platt R.W., Larkin J.C. (2018). Machine learning for fetal growth prediction. Epidemiology.

[6] Ghabri H., Alqahtani M.S., Ben Othman S., Al-Rasheed A., Abbas M., Almubarak H.A., Sakli H., Abdelkarim M.N. (2023). Transfer learning for accurate fetal organ classification from ultrasound images: a potential tool for maternal healthcare providers. Sci. Rep..

[7] Sobhaninia Z., Rafiei S., Emami A., Karimi N., Najarian K., Samavi S., Soroushmehr S.M.R. (2019). Proc. IEEE Eng. Med. Biol. Soc. (EMBC) Annu. Int. Conf..

[8] Hasan S., Akter S. (2023). An improved ensemble model of hyperparameter tuned ML algorithms for fetal health prediction. Int. J. Inform. Technol..

[9] Baumgartner C.F., Kamnitsas K., Matthew J., Fletcher T.P., Smith S., Koch L.M., Kainz B., Rueckert D. (2017). SonoNet: real-time detection and localisation of fetal standard scan planes in freehand ultrasound. IEEE Trans. Med. Imag..

[10] Chowdhury A., Chahar A., Eswara R., Raheem M.A., Ehetesham S., Thulasidoss B.K. (2022). Proc. 2022 8th Int. Conf. Adv. Comput. Commun. Syst. (ICACCS).

[11] Bashir Z. (2025). Clinical validation of explainable AI for fetal growth scans with realtime feedback. Sci. Rep..

[12] Kuzu A., Santur Y. (2023). Early diagnosis and classification of fetal health status from a fetal cardiotocography dataset using ensemble learning. Diagnostics.

[13] Salini Y., Mohanty S.N., Naga V., Yang M., Chalapathi M.M.V.C. (2024). Cardiotocography data analysis for fetal health classification using machine learning models. IEEE Access..

[14] Fiorentino M.C., Villani F.P., Di Cosmo M., Frontoni E., Moccia S. (2023). A review on deep-learning algorithms for fetal ultrasound-image analysis. Med. Image Anal..

[15] Singh K., Shyry P., Franklin R.G. (2023). Efficient fetal health monitoring and classification with machine learning, 2023 7th Int. Conf. Intell. Comput. Control Syst. (ICICCS).

[16] O’Sullivan M., Gabruseva T., Boylan G.B., O’Riordan M., Lightbody G., Marnane W. (2021). Proc. 29th Eur. Signal Process. Conf. (EUSIPCO).

[17] Mandala S.K. (2023). Unveiling the unborn: advancing fetal health classification through machine learning. Artif. Intell. Health..

[18] Deepa D.M.S., Sujithra M. (2024). Classification of fetal health on cardiotocograph data using machine learning techniques. Grenze Int. J. Eng. Technology (GIJET).

[19] Frasch M.G., Strong S.B., Nilosek D., Leaverton J., Schifrin B.S. (2021). Detection of preventable fetal distress during labor from scanned cardiotocogram tracings using deep learning. Front Pediatr..

[20] Mehbodniya A., Lazar A.J.P., Webber J., Sharma D.K., Jayagopalan S., K K., Singh P., Rajan R., Pandya S., Sengan S. (2021). Fetal health classification from cardiotocographic data using machine learning. Expert Syst..

[21] Singh R. (2025). Advancing prenatal healthcare by explainable AI enhanced fetal ultrasound image segmentation using UNet++ with attention. Sci. Rep..

[22] Grünebaum A., Chervenak F.A. (2025). Generative artificial intelligence for counseling of fetal malformations following ultrasound diagnosis. J. Perinat Med..

[23] Burgos-Artizzu X.P., Coronado-Gutierrez D., Valenzuela-Alcaraz B., Bonet-Carne E., Eixarch E., Crispi F., Gratacós E. (2020).

[24] Dr. Sam’s Imaging Library (2023). Obstetric ultrasound normal vs abnormal images | fetal, placenta, umbilical cord pathologies USG, *YouTube*. https://www.youtube.com/watch?v=mJ1qLENEmCU.

[25] Larxel (2020). Fetal health classification, Kaggle. https://www.kaggle.com/datasets/andrewmvd/fetal-health-classification.

[26] Maternal health risk data, Kaggle, 2021. [Online]. Available: https://www.kaggle.com/datasets/csafrit2/maternal-health-risk-data. [Accessed: Feb. 23, 2025].

[27] Mennickent D., Rodríguez A., Opazo M.C., Riedel C.A., Castro E., Eriz-Salinas A., Appel-Rubio J., Aguayo C., Damiano A.E., Guzmán-Gutiérrez E., Araya J. (2023). Machine learning applied in maternal and fetal health: a narrative review focused on pregnancy diseases and complications. Front Endocrin..

[28] Chaurasia P. (2025). Machine learning and explainable artificial intelligence to predict and interpret lead toxicity in pregnant women and unborn baby. Front. Digital Health..

